# 非小细胞肺癌中Ang-2相关研究进展

**DOI:** 10.3779/j.issn.1009-3419.2018.11.09

**Published:** 2018-11-20

**Authors:** 欣 华, 晓莉 朱

**Affiliations:** 1 210000 南京，东南大学医学院 Medical College of Southeast University, Affiliated Zhongda Hospital of Southeast University, Nanjing 210000, China; 2 210000 南京，东南大学附属中大医院呼吸科 Department of Respiration, Affiliated Zhongda Hospital of Southeast University, Nanjing 210000, China

**Keywords:** 肺肿瘤, 血管生成素-2, 新生血管, 侵袭与转移, Lung neoplasms, Angiopoietin-2, Angiogenesis, Invasion and metastasis

## Abstract

非小细胞肺癌（non-small cell lung cancer, NSCLC）是全世界病死率最高的恶性肿瘤之一，也是临床上棘手的难题，其发生发展与肿瘤新生血管的形成密不可分。血管生成素-2（angiopoietin-2, Ang-2）是目前研究较为深入且重要的促血管生成因子，已被证实在NSCLC组织、血液中表达显著上调，并与癌细胞恶性生物学行为呈正相关，有望成为NSCLC诊断和预后的潜在生物学标志物。目前国内外对于Ang-2是如何参与NSCLC进展的相关研究主要集中于增殖、侵袭、转移等方面。本文对近年来NSCLC中涉及Ang-2调控机制的各项研究和文献报道进行总结与评价，期望对未来针对Ang-2的靶向药物研究有所启发。

## Abstract (en)

Non-small cell lung cancer (NSCLC) is one of the malignant tumors with highest mortality in the world, it is still a difficult problem in clinical field. Its occurrence and development are closely associated with tumor angiogenesis. Angiopoietin-2 (Ang-2) is an important angiogenesis factor that has involved in many researches and it has been confirmed that the expression of Ang-2 is significantly up-regulated in tissues and blood of NSCLC. Meanwhile, Ang-2 is related to malignant biological behavior of cancer cells, making it a potential biological marker for the diagnosis and prognosis of NSCLC. At present, researches on Ang-2 how to promote the progression of NSCLC around the world are focused on Ang-2 regulating the proliferation, invasion, and metastasis of NSCLC. This paper summarized and estimated the studies and literature reports of regulatory mechanisms of Ang-2 in NSCLC, hopefully it could help looking for targeted drug treatment of Ang-2 in the future.

肺癌是现阶段发病率及病死率最高的恶性肿瘤之一，其中约85%为非小细胞肺癌（non-small cell lung cancer, NSCLC）^[1]^。一般情况下，NSCLC的发生发展是由多途径、多因素共同作用的结果，其中肿瘤新生血管的形成在肿瘤增殖、侵袭和转移的过程中起到重要作用^[2]^。一方面，肿瘤组织想要生长必须依赖新生血管为其提供血流灌注，给予营养物质，从而增强其增殖和侵袭活力；另一方面，新生血管的内皮通透性高，癌细胞容易突破内层，浸润周围器官和组织，也可利用新生血管通过血液循环将原发癌细胞送至转移的靶器官^[3]^。

肿瘤新生血管形成过程复杂，由多个促进因子、抑制因子共同调控，当促进血管生成的因子作用强于抑制血管生成的因子时，肿瘤新生血管便开始生成。血管生成素-2（angiopoietin-2, Ang-2）是目前研究较为深入且重要的促血管生成因子，已被证实Ang-2在NSCLC组织、血清中异常表达^[4]^，然而Ang-2的表达与癌细胞增殖、侵袭、转移之间的关系仍有待探索，这也对寻找NSCLC治疗的新靶点有重要指导意义。

## 1 Ang-2的生物学作用

### 1.1 Ang家族及Ang-2的生物学作用

血管生成素家族包括Ang-1、Ang-2、Ang-3、Ang-4，它们都可与酪氨酸激酶受体Tie2结合，启动或调控血管生成过程，其中Ang-1、Ang-2与血管生成的关系最为密切。Ang-1在正常组织中表达广泛，与Tie2受体结合后可驱化附近的血管支持细胞如平滑肌细胞和周细胞至血管内皮细胞周围，使内皮细胞和周围支持细胞紧密连接，从而稳定血管，最终起到限制肿瘤新生血管生成、抑制肿瘤生长的作用^[5]^。Makinde等^[6]^发现，Ang-1或Tie2蛋白缺乏均会影响到本身血管的完整性，使小鼠死于胚胎期，证实了Ang-1作为血管稳定因子的重要作用。

Ang-2主要由血管内皮细胞及内皮旁细胞分泌，正常情况下在胎盘、子宫、卵巢中表达^[7]^。在缺氧或炎症反应时会引起Ang-2的异常释放，其作为Ang-1的天然拮抗剂，竞争性地与Tie2结合，从而阻断Ang-1的稳定血管作用，导致内皮细胞与周围细胞的连接离断，引起内皮细胞的不稳定，由此造成血管失稳和内皮活化，在血管内皮生长因子（vascular endothelial growth factor, VEGF）存在的情况下，Ang-2会促进内皮细胞接受VEGF等血管生成诱导剂的出芽信号，使肿瘤组织内的新生血管持续生成^[8]^。目前已发现Ang-2/Tie2系统在多种恶性肿瘤的组织、血液中表达升高，并与肿瘤微血管密度（intratumoral microvessel density, IMVD）、临床分期、预后等关系密切，有望成为重要的抗肿瘤血管形成靶点^[9]^。

### 1.2 Ang-2和VEGF的相互作用

主要的内皮细胞特异性生长因子除了Ang家族外，还有VEGF家族。VEGF是目前最强的促血管生成活性分子，其家族成员包括VEGF-A、VEGF-B、VEGF-C、VEGF-D及其受体VEGFR-1、VEGFR-2、VEGFR-3^[10]^。肿瘤来源的VEGF可促进其本身的分泌，放大促血管生成信号，并可在肺癌细胞系中通过PI3K/AKT、RAS/ERK和STAT3等信号通路诱导Ang-2等各种血管生成因子的显著分泌，形成血管生成因子网络，共同调节肿瘤血管生成^[11]^。肿瘤组织生长到一定体积时，会发生不同程度的缺氧。在常见的肺癌细胞株A549中研究发现，缺氧会刺激细胞分泌缺氧诱导因子（hypoxia inducible factor-1α, HIF-1α）、VEGF、Ang-2的生成，这种表达依赖于PI3K/Akt信号通路途径^[12]^。现已证实^[13]^，Ang-2与VEGF在肿瘤新生血管生成上关系密切，且具有相互促进作用，Ang-2可上调VEGF的表达，而VEGF也可以反过来促进Ang-2的分泌。在VEGF的影响下，Ang-2具有促血管生成或退化的双重作用：一方面，Ang-2本身可诱导肿瘤组织内新生血管呈非出芽式生长，但此时的新生血管周边细胞缺如，胶原排列紊乱，管壁基质稀少，故生成很不稳定，只有在VEGF的作用下，内皮细胞才能发生迁移、增殖，最终形成成熟的新生血管；另一方面，当无VEGF时，Ang-2则诱导原有血管退化与数目减少，同时伴有内皮凋亡^[14]^。

## 2 Ang-2在NSCLC中的异常表达

### 2.1 Ang-2在NSCLC中的诊断价值

为了明确在NSCLC中Ang-2的表达情况，Wong等^[15]^率先在28对肺癌和癌旁组织中验证发现，正常组织中Ang-1和Tie2的表达显著上调，而肺癌组织中VEGF和Ang-2表达显著上调，并且Ang-2表达量与VEGF表达的程度和强度呈显著正相关，这表明在正常情况下Ang-1/Tie2途径介导的血管稳定机制占主导地位，但在肿瘤微环境作用下Ang-2和VEGF被相伴诱导上调，两者相互协作介导肿瘤血管生成。后来陆续有研究^[16-18]^证实NSCLC患者组织、血清中Ang-1表达明显下降，Ang-2、VEGF呈高表达，且肿瘤中这些血管生成分子的表达谱差异与IMVD及肿瘤侵袭、转移等恶性生物学行为显著相关，提示Ang-2及VEGF可以作为NSCLC早期诊断的潜在生物标记物。

为了评价Ang-2的诊断价值，2016年Xu等^[19]^在188例孤立性肺结节（solitary pulmonary nodule, SPN）中检测发现，Ang-2诊断恶性SPN患者和健康志愿者以及恶性SPN和良性SPN患者之间的敏感度和特异度分别为80.5%和97.5%，以及69.5%和92.5%。类似地，在恶性胸腔积液的肺癌患者中Ang-2和VEGF的水平显著升高，检测Ang-2、VEGF、癌胚抗原（carcino-embryonic antigen, CEA）诊断肺癌合并恶性胸腔积液的价值，发现三者均能特异性诊断，当联合诊断效率最高，敏感性达97.7%，特异性达66.7%^[20]^。

### 2.2 Ang-2在NSCLC中的预后价值

目前已有充分研究证实Ang-2可以作为鉴别NSCLC的生物标记物，而且与肿瘤的分期及预后有一定关系。Qiu等^[21]^的最新研究显示，肺癌组织中Ang-2的表达水平与临床分期、肿瘤直径、分化程度有关。2017年一项纳入了1, 911例肺癌病例的荟萃分析^[22]^显示，血清高Ang-2水平与较晚的临床分期（Ⅲ期、Ⅳ期）、淋巴结转移相关，与年龄、性别、吸烟史、组织学类型无关，且高表达的患者预后显著变差，这提示Ang-2在肿瘤侵袭、淋巴性转移方面起到重要作用，提示Ang-2的表达水平可作为肺癌预后预测因子。

Tanaka等^[23]^分析236例NSCLC患者生存率与Ang-2、VEGF表达情况的关系，结果显示：Ang-2阳性较阴性表达的患者5年生存率显著下降（53.5%与70.3%）；Ang-2阳性、VEGF高的患者生存率较Ang-2阳性、VEGF低的患者显著下降（41.4%与63.6%），提示Ang-2阳性导致NSCLC患者总体生存率降低，而且这种影响在VEGF共同高表达时更明显。类似地，Andersen等^[24]^在335例NSCLC病例中发现两者共同高表达时具有显著的差存活率和预后。以上结果可用Ang-2的生物学功能来解释：随着肿瘤体积的增加，当新生血管未形成时，瘤内缺氧导致肿瘤细胞表达Ang-2，从而诱导内皮细胞不稳定，血管通透性增加，不稳定的血管出芽，在高表达的VEGF等生长因子刺激下最终新生血管成熟，促进癌细胞增殖及转移。

## 3 Ang-2促进NSCLC增殖、侵袭、转移的作用机制

肿瘤的发生、发展依赖于新生血管的形成，但Ang-2在NSCLC中是如何作用以促进新生血管生成，继而导致肿瘤增殖、侵袭和转移仍不明确，目前相关研究进展如下。

### 3.1 Ang-2-TEM轴

TEMs（Tie2 expressing monocytes）是指表达Tie2的单核细胞/巨噬细胞，其在慢性炎性疾病、肿瘤血管及淋巴管生成中起到重要作用，研究表明Ang-2可通过调节此亚群，起到调节肿瘤血管生成和免疫系统的作用^[25]^。Coffelt等^[26]^研究发现Ang-2可以募集人TEMs，并上调胸苷磷酸化酶（thymidine phosphorylase, TP）、组织蛋白酶B（cathepsin, CTSB）两种促血管生成酶和VEGF、基质金属蛋白酶9（matrix metalloproteinase, MMP9）、环氧化酶-2（cyclooxygenase, COX-2）和WNT5A等强效促血管生成因子的表达来显著增强TEMs的促血管生成活性。随后，陆续证实了Ang-2还可以通过刺激TEMs而上调强效免疫抑制因子如白细胞介素10（interleukin, IL-10）、人甘露糖受体C1（mannose receptor, MRC1）和调节性T细胞（rgulatory cells, Tregs）的趋化因子配体17（chemokine ligand, CCL17），并下调肿瘤坏死因子α（tumor necrosis factor alpha, TNF-α）、IL-6、IL-12等细胞因子，最终抑制细胞毒性T淋巴细胞（cytotoxic lymphocytes, CTLs）的活化并扩展CD4^+^CD25^+^FOXP3^+^Tregs的表达^[27-30]^。随后发现，高水平的IL-10、MRC1、CCL17以及低水平的TNF-α、IL-6、IL-12细胞因子表达属于巨噬细胞“M2”激活状态下的产物^[31]^，因此推测肿瘤中的Ang-2通过诱导TEMs表达“M2”表型以发挥促血管生成作用。值得注意的是，Ang-2对肿瘤血管的作用具有器官依赖性。在小鼠肝转移模型中，敲减Ang-2后，血清中粒细胞集落刺激因子（granulocyte-colony stimulating factor, G-CSF）和CXC趋化因子配体1（CXC chemokine ligand, CXCL1）显著上调，导致Tie2^+^/CD11b^+^/CD31^-^型免疫细胞的募集，最终肿瘤血管代偿性生成；相反，在肺转移瘤中Ang-2缺陷型小鼠肿瘤血管生成减少，血清G-CSF不增加，但发现转移过程中伴随着细胞因子IL-13、IL-15的显著增加，而IL-13已知可抑制某些癌细胞系的增殖，IL-15可启动T细胞分裂、NK细胞发育和CD8^+^记忆T细胞的产生^[32]^。因此，这些细胞因子的差异性变化可以解释Ang-2对肺和肝中的肿瘤生长以及血管生成的不同作用。

### 3.2 Ang-2/Tie2通路中涉及的相关细胞因子和蛋白

#### 3.2.1 IL-10

IL-10是一种与NSCLC预后相关的免疫抑制因子，Hatanaka等^[33]^在95例NSCLC手术标本中验证出IL-10的高表达与*Ang-2*和*Tie2*基因的表达显著相关，且在IL-10高表达区域肿瘤血管的数量明显增多，从而提示了Ang-2/Tie2系统网络可通过诱导免疫因子IL-10的表达实现对新生血管的调控。

#### 3.2.2 血管渗漏相关因子

在肺癌转移前期，已有研究^[34]^证明Ang-2、MMP3和MMP10表达上调，继而导致血管通透性增加，循环肿瘤细胞外渗和转移，进一步研究证实，肿瘤细胞的恶性程度与其诱导Ang-2、MMP3和MMP10的表达能力呈正相关，且TNF-α和转化生长因子-β（transforming growth factor-β, TGF-β）也参与了这些转移相关因子的调节。此外，Ang-2还通过促进磷脂酰肌醇特异性磷酯酶（phosphatidylinositol specific phospholipase, PLCγ2）的激活和钙离子（Ca^+^）内流等生物学行为以诱导多种血管渗漏相关细胞因子（组胺、缓激肽、VEGF）的表达^[35]^，表明Ang-2可通过调节血管渗漏过程促进肿瘤的发展。

#### 3.2.3 中性粒细胞胞外诱捕网（neutrophil extracellular traps, NETs）

NETs是中性粒细胞在适宜刺激下释放到胞外的一种纤维网格样结构。最初被认为具有捕获细菌、控制感染的作用，近年来NETs被发现还具有细胞毒性和免疫原性成分，与多种非感染性病理状态如肿瘤、自身免疫疾病相关。Lavoie等^[36]^证实，Ang-1和Ang-2均可与NETs表面的Tie2受体结合，并上调NETs的合成和释放，释放后的NETs可诱导中性粒细胞和内皮细胞活化，从而在Ang-2介导的促炎和促血管生成过程中作出贡献。

#### 3.2.4 凋亡、转移相关蛋白

HMGA2是一种与肿瘤发展相关的迁移蛋白，2016年一项研究^[37]^发现在肺鳞癌中Ang可结合其下游调控蛋白HMGA2的启动子，使其表达量增加，继而显著提高了细胞的增殖、迁移及侵袭能力。Survivin作为迄今发现的功能最强的凋亡抑制蛋白，其在肺癌患者血清中表达量高于正常对照组，且与Ang-2和VEGF的表达呈显著正相关，有研究^[38]^表明在VEGF的影响下，Ang-2可上调survivin的表达从而促进肿瘤血管生成，三者协同促进肺癌的进展和转移。肺癌细胞中金属蛋白酶9（A disintegrin and metalloprotease, ADAM9）的上调与大脑转移显著相关，研究显示在ADAM9沉默的细胞中Ang-2、VEGF-A的表达受到抑制，表明ADAM9可通过血管重构促进肿瘤转移^[39]^。总之，以上研究成果证明Ang-2通过调控各种凋亡、转移蛋白来影响肺癌细胞的生物学行为。

### 3.3 Ang-2与肿瘤炎症微环境

最新的一项荟萃分析^[8]^显示，Ang-2与炎症标志物（纤维蛋白原、白细胞、超敏C反应蛋白）均呈正相关，提示Ang-2在炎症反应中起到了重要作用。转移生态位模型认为，肿瘤发生转移之前，转移位点必须提供有利于肿瘤细胞生长的炎症微环境（即转移生态位），这是形成新的转移灶的前提条件^[40]^。Srivastava等^[41]^在乳腺癌肺转移的模型中发现，肺转移瘤来源的细胞为趋化因子受体2（chemokine receptor, CCR2）阳性和Tie2阴性的转移相关巨噬细胞（CCR2^+^Tie2^-^MAMs），深入研究发现，在转移发生之前，Ang-2可通过自分泌的方式刺激转移生态位处的血管内皮细胞，继而诱导粘附分子-1（intercellular cell adhesion molecule, ICAM-1）和CCL2的表达上调，营造出适合巨噬细胞存活和粘附的生态环境，使得远端气管内皮细胞对CCR2^+^Tie2^-^MAMs粘附敏感从而促进肿瘤转移。Fiedler等^[42]^也同样证实了Ang-2可发挥促炎细胞因子的作用，其通过促进内皮细胞上调ICAM-1和血管细胞粘附分子-1（vascular cell adhesion molecule, VCAM-1）的表达，从而诱导炎性细胞与内皮细胞的牢固粘附。随后Scholz等^[43]^利用体外实验证明了Ang-2诱导的炎性粘附仅对单核细胞有效，而对淋巴细胞没有影响。总之，目前研究均证实了Ang-2可作为造成肿瘤炎症微环境的炎症反应调节剂，参与肿瘤转移和生长的过程。

### 3.4 周细胞的覆盖与EMT

周细胞是血管重要的结构和功能组分，位于血管内皮细胞周围，目前认为周细胞覆盖率高的患者，血管屏障稳定，总体生存率更高^[44]^。周细胞具有抑制或促进肿瘤生长和转移的阶段特异性作用，这种相反的功能受到Ang/Tie2传导信号的调控^[45]^。早期非缺氧肿瘤中，检测到Ang-1的高表达，而Ang-2无明显改变，此时在Ang-1/Tie2的作用下，周细胞增加对血管的包裹，从而提高了血管稳定性，导致“新生血管生成抑制”的表型，阻碍了肿瘤生长和转移；相反，晚期肿瘤负荷增加时，此时Ang-2水平升高，在Ang-2的主导作用下，周细胞覆盖率下降，导致血管缺陷，瘤内缺氧加重，引起上皮间质转化（epithelial mesenchymal transition, EMT）增加，最终促进肿瘤转移^[4, 46]^。

### 3.5 Ang-2其他相关信号通路

#### 3.5.1 钙调磷酸酶-NFAT-Ang-2信号通路

在肿瘤转移之前VEGF即与转移灶处内皮细胞上的VEGF受体结合活化，继而激活PLCγ2/Ca ^2+^途径，继而导致细胞内Ca^2+^上调，使得Ca^2+^调节的钙调磷酸酶被激活，导致活化T细胞核因子（nuclear factor of activated T cells, NFAT）家族的转录因子去磷酸化^[47]^，继而下游的靶标Ang-2增加，之后激活转移灶内皮，促进转移，当过表达内源性抑制剂例如Dscr-1基因后，Ang-2 mRNA显著下调，并抑制了早期转移病灶中内皮活化^[48]^，证实了钙调磷酸酶-NFAT-Ang-2信号通路可调节早期转移灶的内皮活化，在促进转移灶处的肿瘤细胞接种和扩增中起重要作用。

#### 3.5.2 Ang-2-β1-整合素信号通路

β1-整合素在维持内皮肌动蛋白细胞骨架中具有双重的作用，依赖于Ang-2的亚细胞定位功能：细胞外周中的β1-整合素维持肌动蛋白细胞骨架，而细胞中心的β1-整合素拉伸粘连，激活肌动蛋白应力纤维。Ang-2可通过直接相互作用激活内皮β1-整联素，血清中的促炎细胞因子IL-6、IL-1β和TNF-α等也可激活下游的β1-整合素，使得定位在细胞中心的β1-整合素伸长粘连作用显著增强，继而含VE-钙粘连蛋白（VE-cadherin）的肺微血管内皮细胞-细胞连接减弱，最终降低内皮单层完整性，导致屏障功能降低，毛细血管渗漏增加，以此通过Ang-2-β1-整合素信号通路途径促进肿瘤侵袭和转移^[49]^。

### 3.6 Ang-2在淋巴管生成方面的作用

淋巴转移是NSCLC扩散的重要途径，在肿瘤缺氧、高压的微环境作用下，肿瘤细胞和间质细胞会诱导淋巴管生长因子的分泌和活化，而最终淋巴管生成依赖于多种生长因子（包括VEGF家族、Ang家族等）的调节。NSCLC组织、血液中检测到高表达的Ang-2与淋巴结转移和患者低生存率相关，但其具体机制尚未阐明。事实上，Ang-2虽然通过竞争性拮抗作用破坏血管内皮稳定，但在淋巴内皮中Ang-2/Tie2通路发挥的却是激动作用，Ang-2可通过激活淋巴管内皮细胞（lymphatic endothelial cells, LECs）上的Tie2受体，引起LECs的迁移、增殖和淋巴管增生^[50]^。Souma等^[51]^在小鼠模型中发现这种相反的生物学功能取决于血管内皮蛋白酪氨酸磷酸酶（vascular endothelial protein tyrosine phosphatase, VEPTP）的存在与否：LECs中缺乏VEPTP，此时Ang-2/Tie2发挥内皮激动功能，相反，血管内皮表达高水平的VEPTP，而VEPTP会提高Tie2的激活阈值并阻止Ang-2的激活，这揭示了Ang-2促进淋巴转移的部分机制。

## 4 针对Ang-2的靶向治疗进展

肿瘤所处的缺氧、酸性、高间质压力的微环境，形成了药物递送到肿瘤组织中的病理性屏障^[52]^。传统的抗血管生成方法主要集中在抑制新血管形成和破坏原有肿瘤血管方面，代表药物如贝伐珠单抗（抗VEGF单克隆抗体）虽然短期内可抑制肿瘤血管生成，但随后会因血管退化和稀疏造成肿瘤组织的极度缺氧，导致Ang-2的表达上调，继而促进VEGF非依赖性的肿瘤血管生成，并最终导致耐药性增加^[53]^。

多项研究证明，在肿瘤进展早期阶段对VEGF或Ang-2进行阻断可显著减少血管生成、肿瘤生长、侵袭和转移，且减少了血管渗漏和周细胞脱落^[54]^。但单独阻断Ang-2或VEGF表达的效果都不甚满意^[55]^。Ang-2和VEGF双重阻断的抑瘤功效被证明大于单一阻断Ang-2或VEGF，然而，无论是单一或是双重阻断仍然存在增加肿瘤缺氧和随后恶化肿瘤微环境的风险^[56, 57]^。最近在小鼠模型中研究发现，ABTAA（Ang-2抑制和Tie2激活抗体）和ABA（Ang-2抑制抗体）相比，ABTAA诱导肿瘤脉管系统正常化，导致血液灌注增加，且显著减少乳酸酸中毒和肿瘤生长、转移，最终通过营造有利的肿瘤微环境以增强化疗药物的递送^[58]^，可作为有效抗癌药物。因此，未来抗血管生成靶向药物可着力于Ang-2相关因子的双靶点或多靶点联合，但怎样搭配才能发挥最大效能仍在探索，同时寻找实现肿瘤血管正常化、不恶化肿瘤微环境以增强化疗药物递送的靶向药物仍是关键。

## 5 小结与展望

Ang-2作为一类重要的促血管生成因子，已被证实在NSCLC组织、血液中表达显著上调，且与肿瘤TNM分期、淋巴结转移相关，提示Ang-2可作为NSCC诊断和预后的潜在生物学标志物。目前关于Ang-2是如何促进肿瘤增殖、侵袭、转移的相关机制仍在研究，已知其可通过干扰血管内皮稳定性、诱导新生血管生成、调节炎症和血管渗漏过程、促进周细胞脱落、促进淋巴管增生、淋巴内皮细胞增殖等方面实现对癌细胞恶性生物学行为的调控，以上机制为针对Ang-2的靶向药物研究提供新目标。

## References

[1] Zappa C, Mousa SA (2016). Non-small-cell lung cancer: current treatment and future advances. Transl Lung Cancer Res.

[2] Janning M, Loges S (2018). Anti-angiogenics: their value in lung cancer therapy. Oncol Res Treat.

[3] Sanmartín E, Sirera R, Usó M (2014). A gene signature combining the tissue expression of three angiogenic factors is a prognostic marker in early-stage non-small cell lung cancer. Ann Surg Oncol.

[4] Dong Z, Chen J, Yang X (2018). Ang-2 promotes lung cancer metastasis by increasing epithelial-mesenchymal transition. Oncotarget.

[5] Shim WS, Ho IA, Wong PE (2007). Angiopoietin: a TIE(d) balance in tumor angiogenesis. Mol Cancer Res.

[6] Makinde T, Agrawal DK (2008). Intra and extravascular transmembrane signalling of angiopoietin-1-Tie2 receptor in health and disease. J Cell Mol Med.

[7] Zhou M, Qu ZL, Bai JP (2016). Expression of Tie2, Ang1, and Ang2 Proteins in hemangioma and vascular malformation. Hua Zhong Ke Ji Da Xue Xue Bao (Yi Xue Ban).

[8] Schuldt EA, Lieb W, Dörr M (2018). Circulating angiopoietin-2 and its soluble receptor Tie-2 concentrations are related to inflammatory markers in the general population. Cytokine.

[9] Rigamonti N, Kadioglu E, Keklikoglou S (2014). Role of angiopoietin-2 in adaptive tumor resistance to VEGF signaling blockade. Cell Rep.

[10] Reck M, Garassino MC, Imbimbo M (2018). Antiangiogenic therapy for patients with aggressive or refractory advanced non-small cell lung cancer in the second-line setting. Lung Cancer.

[11] Frezzetti D, Gallo M, Roma C (2016). Vascular endothelial growth factor a regulates the secretion of different angiogenic factors in lung cancer cells. J Cell Physiol.

[12] Xie Y, Qi Y, Zhang Y (2017). Regulation of angiogenic factors by the PI3K/Akt pathway in A549 lung cancer cells under hypoxic conditions. Oncol Lett.

[13] Xing WT, Song FQ, Hu WC (2015). The expression and significance of VEGF and Ang-2 in serum of osteosarcoma patients. Xian Dai Zhong Liu Yi Xue.

[14] Lobov IB, Brooks PC, Lang RA (2002). Angiopoietin-2 displays VEGF-dependent modulation of capillary structure and endothelial cell survival *in vivo*. Proc Natl Acad Sci U S A.

[15] Wong MP, Chan SY, Fu KH (2000). The angiopoietins, tie2 and vascular endothelial growth factor are differentially expressed in the transformation of normal lung to non-small cell lung carcinomas. Lung Cancer.

[16] Liang YM, Zhang PF, Liu J (2017). Diagnostic specificity and prognostic judgment accuracy of surum VEGF and Ang-2 on non-small cell lung cancer. Lin Chuang Fei Ke Za Zhi.

[17] Gao XL (2012). Expression and correlation of serum Ang-1 and Ang-2 in patients with lung cancer. Zhongguo Xue Ye Liu Bian Xue Za Zhi.

[18] Abudoushalamu Abudoureyimu, Yang J, Aili Saiding (2017). Expression of Ang-2 and HSP90α in serum of patients with non-small cell cancer and its correlation with TNM staging. Guo Ji Jian Yan Yi Xue Za Zhi.

[19] Xu C, Wang W, Wang Y (2016). Serum angiopoietin-2 as a clinical marker for lung cancer in patients with solitary pulmonary nodules. Ann Clin Lab Sci.

[20] Zhang L, Liu H (2017). Combined determination of angiopoietin-2, vascular endothelial growth factor and carcinoembryonic antigen in pleural effusion of patients with lung cancer. Jian Yan Yi Xue.

[21] Qiu LW, Chen JR, Yang XL (2018). Abnormal expression of angiopoietin-2 associated with invasion, metastasis and prognosis of lung cancer. Zhonghua Yi Xue Za Zhi.

[22] Xu Y, Zhang Y, Wang Z (2017). The role of serum angiopoietin-2 levels in progression and prognosis of lung cancer: A *meta*-analysis. Medicine (Baltimore).

[23] Tanaka F, Ishikawa S, Yanagihara K (2002). Expression of angiopoietins and its clinical significance in non-small cell lung cancer. Cancer Res.

[24] Andersen S, Donnem T, Al-Shibli K (2011). Prognostic impacts of angiopoietins in NSCLC tumor cells and stroma: VEGF-A impact is strongly associated with Ang-2. PLoS One.

[25] Varricchi G, Loffredo S, Galdiero MR (2018). Innate effector cells in angiogenesis and lymphangiogenesis. Curr Opin Immunol.

[26] Coffelt SB, Tal AO, Scholz A (2010). Angiopoietin-2 regulates gene expression in TIE2-expressing monocytes and augments their inherent proangiogenic functions. Cancer Res.

[27] Coffelt SB, Chen YY, Muthana M (2011). Angiopoietin 2 stimulates TIE2-expressing monocytes to suppress T cell activation and to promote regulatory T cell expansion. J Immunol.

[28] Murdoch C, Tazzyman S, Webster S (2007). Expression of Tie-2 by human monocytes and their responses to angiopoietin-2. J Immunol.

[29] Mills CD, Kincaid K, Alt JM (2017). Pillars article: M-1/M-2 macrophages and the Th1/Th2 paradigm. J Immunol.

[30] Palazon A, Tyrakis PA, Macias D (2017). An HIF-1α/VEGF-A Axis in cytotoxic T cells regulates tumor progression. Cancer Cell.

[31] Turrini R, Pabois A, Xenarios I (2017). TIE-2 expressing monocytes in human cancers. Oncoimmunology.

[32] Im JH, Tapmeier T, Balathasan L (2013). G-CSF rescues tumor growth and neo-angiogenesis during liver metastasis under host angiopoietin-2 deficiency. Int J Cancer.

[33] Hatanaka H, Abe Y, Naruke M (2001). Significant correlation between interleukin 10 expression and vascularization through angiopoietin/TIE2 networks in non-small cell lung cancer. Clin Cancer Res.

[34] Huang Y, Song N, Ding Y (2009). Pulmonary vascular destabilization in the premetastatic phase facilitates lung metastasis. Cancer Res.

[35] Benest AV, Kruse K, Savant S (2013). Angiopoietin-2 is critical for cytokine-induced vascular leakage. PLoS One.

[36] Lavoie SS, Dumas E, Vulesevic B (2018). Synthesis of human neutrophil extracellular traps contributes to angiopoietin-mediated *in vitro* proinflammatory and proangiogenic activities. J Immunol.

[37] Xu L (2016). ANG promotes proliferation and invasion of the cell of lung squamous carcinoma by up-regulating HMGA2. Di Er Jun Yi Da Xue Xue Bao.

[38] Zhai NL, Liu JP, Gao FQ (2016). Expression of serum VEGF, Ang-2 and survivin in lung carcinoma and their correlation. Binzhou Yi Xue Yuan Xue Bao.

[39] Lin CY, Cho CF, Bai ST (2017). ADAM9 promotes lung cancer progression through vascular remodeling by VEGFA, ANGPT2, and PLAT. Sci Rep.

[40] Qian JJ, Akçay E (2018). Competition and niche construction in a model of cancer metastasis. PLoS One.

[41] Srivastava K, Hu J, Korn C (2014). Postsurgical adjuvant tumor therapy by combining anti-angiopoietin-2 and metronomic chemotherapy limits metastatic growth. Cancer Cell.

[42] Fiedler U, Reiss Y, Scharpfenecker M (2006). Angiopoietin-2 sensitizes endothelial cells to TNF-alpha and has a crucial role in the induction of inflammation. Nat Med.

[43] Scholz A, Plate KH, Reiss Y (2015). Angiopoietin-2: a multifaceted cytokine that functions in both angiogenesis and inflammation. Ann N Y Acad Sci.

[44] Zhao H, Darden J, Chappell JC (2018). Establishment and characterization of an embryonic pericyte cell line. Microcirculation.

[45] Teichert M, Milde L, Holm A (2017). Pericyte-expressed Tie2 controls angiogenesis and vessel maturation. Nat Commun.

[46] Keskin D, Kim J, Cooke VG (2015). Targeting vascular pericytes in hypoxic tumors increases lung metastasis via angiopoietin-2. Cell Rep.

[47] Rozen EJ, RoewenstrunkK J, Barallobre MJ (2018). DYRK1A kinase positively regulates angiogenic responses in endothelial cells. Cell Rep.

[48] Minami T, Jiang S, Schadler K (2013). The calcineurin-NFAT-angiopoietin-2 signaling axis in lung endothelium is critical for the establishment of lung metastases. Cell Rep.

[49] Hakanpaa L, Kiss EA, Jacquemet G (2018). Targeting β1-integrin inhibits vascular leakage in endotoxemia. Proc Natl Acad Sci U S A.

[50] Zheng W, Aspelund A, Alitalo K (2014). Lymphangiogenic factors, mechanisms, and applications. J Clin Invest.

[51] Souma T, Thomson BR, Heinen S (2018). Context-dependent functions of angiopoietin 2 are determined by the endothelial phosphatase VEPTP. Proc Natl Acad Sci U S A.

[52] Gatenby RA, Gillies RJ (2004). Why do cancers have high aerobic glycolysis?. Nat Rev Cancer.

[53] Fukumura D, Kloepper J, Amoozgar Z (2018). Enhancing cancer immunotherapy using antiangiogenics: opportunities and challenges. Nat Rev Clin Oncol.

[54] Daly C, Eichten A, Castanaro C (2013). Angiopoietin-2 functions as a Tie2 agonist in tumor models, where it limits the effects of VEGF inhibition. Cancer Res.

[55] Wang J, Chen J, Guo Y (2017). Strategies targeting angiogenesis in advanced non-small cell lung cancer. Oncotarget.

[56] Reardon DA, Lassman AB, Schiff D (2018). Phase 2 and biomarker study of trebananib, an angiopoietin-blocking peptibody, with and without bevacizumab for patients with recurrent glioblastoma. Cancer.

[57] Jain RK (2014). Antiangiogenesis strategies revisited: from starving tumors to alleviating hypoxia. Cancer Cell.

[58] Park JS, Kim IK, Han S (2016). Normalization of tumor vessels by Tie2 activation and Ang2 inhibition enhances drug delivery and produces a favorable tumor microenvironment. Cancer Cell.

